# Fatal Aortic Dissection in a Patient with Giant Cell Arteritis: A Case Report and Review of the Literature

**DOI:** 10.1155/2013/590721

**Published:** 2013-02-26

**Authors:** Anjeli K. Nayar, Michael Casciello, Jennifer N. Slim, Ahmad M. Slim

**Affiliations:** ^1^Department of Cardiology, San Antonio Military Medical Center, San Antonio, TX 78234, USA; ^2^Sentara Cardiology, Williamsburg, VA 23188, USA; ^3^Department of Medicine, San Antonio Military Medical Center, San Antonio, TX 78234, USA

## Abstract

Giant cell arteritis may lead to catastrophic, large-vessel complications from chronic vascular wall inflammation without prompt diagnosis and treatment. We describe a rare case of acute aortic dissection without preceding aneurysm secondary to histologically confirmed giant cell arteritis (GCA) in an 85-year-old female with a four-year history of polymyalgia rheumatica and temporal arteritis diagnosed per biopsy six months prior to presentation. The literature is reviewed and the clinical implications of this case are discussed.

## 1. Case Report

An 85-year-old woman with a four-year history of polymyalgia rheumatica and temporal arteritis diagnosed per biopsy six months prior to presentation with an acute sensation of neck tightness with radiation to her bilateral shoulders and the epigastrium. Past medical history was significant for atrial fibrillation status following ablation, coronary artery disease status following remote myocardial infarction medically managed, hyperlipidemia, and hypertension. Her home medications included atenolol, coumadin, and prednisone 20 mg PO twice daily. She had intermittently received several rapidly tapered oral steroid courses over the preceding two years for polymyalgia rheumatica and temporal arteritis flares with resolution of clinical symptoms and normalization of acute inflammatory markers. 

 At admission, her vital signs and cardiopulmonary exam were within normal limits. An electrocardiogram showed sinus rhythm with first-degree atrioventricular block. Cardiac biomarkers and a chest radiograph were unremarkable. Labs were significant for a stable known normochromic normocytic anemia of chronic disease (hemoglobin 10.3 g/dL), indolent chronic lymphocytic leukemia (white blood cell count of 11,400), an international normalized ratio of 2.0, and an erythrocyte sedimentation rate (ESR) of 49 mm/hr. A transthoracic echocardiogram eight months prior to admission demonstrated hyperdynamic left ventricular function with ejection fraction of 65%–70%, mild aortic insufficiency, and mild concentric left ventricular hypertrophy.

 Within several hours of admission, the patient was found to be unresponsive and progressively bradycardic with a depressed left ventricular function with ejection fraction of 45%. She later developed a wide complex tachycardia before terminating in pulseless electrical activity.

 Subsequent autopsy showed extensive granulomatous inflammation with lymphocytes, giant cells, and elastic membrane destruction in the aorta and vertebrobasilar and coronary arteries. There was evidence of early aortic root and proximal segment dissection without aneurysmal dilatation, associated with a mild pericardial effusion and a left hemothorax. Despite mild, nonobstructive atherosclerosis, there was no evidence of coronary artery occlusion or myocardial focal ischemic changes. Additionally, no large artery stenosis was appreciated.

## 2. Discussion

Giant cell arteritis (GCA) predominantly affects people of Northern European descent and has an annual incidence of 20 cases per 100,000 persons aged greater than fifty years [1, 2]. The risk of developing GCA increases with age with a male-to-female ratio of 1 : 2-3; it is twenty times more common among nonagenarians than those in their fifth or sixth decade [3, 4]. The average age of patients at time of aortic dissection is 74.5 years (range 58–87 years old) with a similar female predominance of 1 : 4 [3]. In contrast, the typical patient suffering from a non-GCA associated aortic dissection is two to five times more likely to be male, with peak occurrence at 50–66 years old [5]. Improved detection and new imaging modalities have likely contributed to the apparent increase in incidence of large-artery complications over the decades.

The pathogenesis of GCA is unknown [1] but is theorized to be an antigen-driven disease characterized by a granulomatous inflammatory reaction in the adventitia of medium and large arteries [4]. Some genetic polymorphisms, such as HLA-DRB1*-04 alleles, which regulate the expression of cytokines and T cells may contribute to GCA susceptibility [6, 7]. Activated macrophages and CD4+ T-lymphocytes stimulated by antigen-presenting cells in the vasa vasorum produce inflammatory mediators and reactive oxygen intermediates that disrupt the internal elastic lamina. Resultant neointimal arterial lesions subsequently signal the migration of smooth muscle cells leading to intimal hyperplasia and ultimately ischemic manifestations. It is hypothesized that thoracic involvement of GCA may be more common than abdominal due to its relatively greater vasa vasorum. 

There is a wide heterogeneity of large-vessel GCA presentations. Additionally, significant seasonal variations have been observed [8]. The most common clinical features include new onset atypical and severe headache (60%–90%), temporal artery tenderness (40%–70%), fever (20%–50%), polymyalgia rheumatica (30%–50%), claudication (jaw, 30–70% and extremity, 5%–15%), and visual changes (20%) [1, 2]. Acute symptomatic aneurysm dissection or clinically apparent aortitis is a presenting feature in ≥15% [1]. However, symptoms may occur late, if any, and are likely underrecognized.

While the most common predisposing factor for aortic dissection is hypertension [4, 9], other risk factors may include tobacco smoking and menopausal, hypoestrogenic states that result in asymmetric smooth muscle cell loss [10]. Nuenninghoff et al. identified hyperlipidemia and coronary artery disease as predictors of aortic aneurysm or dissection, although atherosclerosis itself has not been shown to precipitate GCA [14]. Up to 15% of people with polymyalgia rheumatica will develop giant cell arteritis [11], and conversely, approximately 30%–50% of patients with GCA will have features of PMR [12].

The extent of large artery involvement of GCA is uncertain, but clinically apparent large-vessel disease comprises an estimated 9%–14% of cases [13] with an incidence of aortic aneurysm with or without dissection of approximately 18 per 1000 person-years [14, 15]. Clinically occult GCA has been demonstrated in almost 50% of patients before aortic dissection [4, 16], a complication with an incidence of approximately 2–4 per 100,000 per year [17, 18]. In a retrospective cohort study by Nuenninghoff et al. in patients diagnosed with GCA over 50 years, only 9 pts (5%) developed aortic dissection without preceding aneurysm which was fatal in 77% of cases [14]. The study by Liu et al. [3] demonstrated that 46% of patients with histopathology-confirmed giant cell arteritis initially presented with aortic dissection, 85% of which cases involved the proximal aorta, resulting in an 80% 2-week mortality rate with fatal pericardial tamponade affecting 50% of these patients [9, 19]. This is similar to the 65%–75% mortality rate for untreated aortic dissection, regardless of etiology [20, 21].

Compared to the general population, patients with GCA are 17.3 and 2.4 times more likely to develop a thoracic or abdominal aneurysm, respectively [1]. Furthermore, approximately 50% of those with thoracic aortic aneurysms will die secondary to associated complications [1]. Other large-vessel complications include upper extremity stenosis (18%–21%) and cerebrovascular ischemic events (7%). Myocardial infarction from GCA is rarely appreciated [1, 22–24]. These statistics emphasize the dramatic consequences and mortality implications of GCA large-vessel extension for which clinical risk should be assessed early in the disease course.

The American College of Rheumatology diagnostic criteria for GCA include (1) age greater than 50 years, (2) recent-onset localized headache, (3) temporal artery tenderness or pulse attenuation, (4) ESR greater than 50 mm/h, and (5) necrotizing vasculitis on arterial biopsy [25]. At least three positive criteria confer greater than 90% sensitivity and specificity for GCA. Among older patients with large-vessel inflammatory involvement, peripheral pulses should also be carefully evaluated to appreciate new bruits, pulses, and symmetry in blood pressure [1]. However, the diagnostic gold standard is histopathological per arterial wall biopsy that may only be 50–80% positive based on the clinical pattern of disease [1]. It has been estimated that the median time from diagnosis of GCA to detection of thoracic aortic dissection is 1.1 yrs [14]. Given the potential morbidity of delayed treatment, diagnosis and immediate therapy should be based on high clinical suspicion [26]. Classic laboratory findings include normocytic anemia and reactive thrombocytosis. Albumin may be moderately decreased, and 25%–35% of patients will have increased aspartate aminotransferase and alkaline phosphatase elevation, findings that normalize with steroid treatment. The erythrocyte sedimentation rate is often more than 100 mm/h, though disease activity may better correlate with C-reactive protein (CRP) levels.

Early diagnosis with various imaging modalities may also facilitate prompt treatment to avoid long-term complications. As per guidelines, the initial evaluation of giant cell arteritis should include computed tomographic or magnetic resonance imaging of the thoracic aorta and branch vessels to assess aneurysm or occlusive disease [2]. Alternatively, the 18-fluorodeoxyglucose positron emission tomography (18-FDG PET) has been proposed as both a sensitive and specific diagnostic test that confirms an active vascular process when clinical symptoms and inflammatory markers are ambiguous or equivocal [27, 28]. In patients with confirmed or suspected temporal arteritis or polymyalgia rheumatica (PMA), PET was positive in 76% of patients with confirmed disease, in 23% with suspected disease, and in 2% of healthy controls, demonstrating the low rate of false positivity [29]. Suggested screening for aneurysms in patients with GCA includes baseline and annual abdominal ultrasound, chest radiography, and transthoracic echocardiography [28]. 

Corticosteroids are first-line treatment, and the time elapsed before initiation is an important prognostic factor. Evidence supports a greater than 50% versus 6% improvement in vision if therapy is administered within 24 hours from symptom onset [30]. The guidelines recommend high dose corticosteroids, such as prednisone 40–60 mg or its equivalent, for initial treatment, though recent evidence supports similar efficacy with 30 to 40 mg daily dose [2, 31]. Split dosing has not demonstrated increased efficacy, and alternate day treatments have resulted in breakthrough symptoms without decreased risk of osteoporosis [32–34]. Approximately 2-3 months after therapy initiation [2], once the disease is adequately controlled, steroid tapering is appropriate and maintenance therapy may continue for years [35]. At this time, there are no evidence-based recommendations to guide steroid therapy after aortic dissection [4]. Regarding adjunctive therapy, clinical trials have not shown cytotoxic and immunosuppressive agents to be effective steroid-sparing agents [36, 37]. However, low-dose aspirin is recommended for its 3-4-fold reduction in associated cranial ischemic events as evidenced in retrospective trials [38, 39].

Given the risk of steroid-induced osteoporosis, bisphosphonates, calcium, and vitamin D supplements may also be considered. Although the guidelines do not include specific surgical recommendations for GCA associated thoracic aorta aneurysms and dissection, patients with symptoms suggestive of thoracic aneurysm expansion or rupture should be promptly evaluated for surgical intervention barring limited life expectancy from comorbidities or a substantially impaired quality of life [2].

## 3. Conclusion

 This case provides a rare histological example of spontaneous aortic dissection secondary to giant cell arteritis without a preceding aneurysm and supports the hypothesis that inadequate treatment of GCA or PMR may predispose to development of aortic dissection [4, 40]. Aortic complications of GCA can be sudden and catastrophic, occurring years after symptomatic resolution [19]. A normal erythrocyte sedimentation rate (ESR) or C-reactive protein (CRP) in treated patients with PMR or GCA does not exclude the possibility of persistent aortic inflammation and associated risk of large-vessel dissection and rupture. Diagnosis and immediate corticosteroid treatment should be based on high clinical suspicion, and surveillance imaging should follow initial assessment for large-vessel involvement [41]. At this time, surgical treatment of asymptomatic aneurysm and suspected dissection is as per atherosclerotic guidelines [28]. However, further research is indicated regarding the surveillance and treatment of aortic aneurysms and dissections in patients with GCA.
